# Gut microbiota and risk of cervical cancer: a Mendelian multivariable randomization study

**DOI:** 10.1007/s12672-025-03717-3

**Published:** 2025-10-17

**Authors:** Lihuan Lu, Zhengqi Li, Ping Qiang, Yang Shao

**Affiliations:** 1 Department of Obstetrics and Gynecology, Zhangjiagang Hospital Affiliated to Soochow University, Zhangjiagang, 215600 Jiangsu China; 2 Department of Gastroenterology, Zhangjiagang Second People’s Hospital, Zhangjiagang, 215600 Jiangsu China

**Keywords:** Gut microbiota, Cervical cancer, Two-sample Mendelian randomization

## Abstract

**Background and hypothesis:**

Epidemiological evidence demonstrates associations between gut microbial dysbiosis and cervical cancer, though causal inference remains limited by potential confounding.

**Study design:**

A meta-analysis of the largest available genome-wide association study (GWAS) meta-analysis using the MiBioGen consortium (n = 14,306 individuals; 8,107,040 SNPs analyzed) was conducted for the summary statistics of the gut microbiome. A two-sample Mendelian randomization study was performed using the statistics of cervical cancer from BioBank Japan (BBJ) and the European Bioinformatics Institute (EBI) GWAS Catalog. The causal relationship between the gut microbiome and cervical cancer was examined using inverse variance weighting, maximum likelihood, MR-Egger, weighted median, weighted model, and MR-PRESSO methods. The Cochran Q statistic was used to quantify the heterogeneity of the instrumental variables.

**Study results:**

The odds ratio (OR) values obtained by the IVW method indicate that the consistent microbial communities in the validation results from two different cervical cancer datasets are: Actinomyces (BBJ OR = 0.52, 95% CI: 0.29–0.92, P < 0.05), (EBI OR = 0.55, 95% CI: 0.29–0.87, P < 0.05) It has a protective effect on the occurrence of cervical cancer, Lachnospiraceae UCG001 (BBJ OR = 2.00, 95% CI: 1.11–3.58, P < 0.05), ( EBI OR = 1.91, 95% CI: 1.16–3.13, P < 0.05) It has a promoting risk effect on the occurrence of cervical cancer, and there is no significant heterogeneity or horizontal pleiotropy.

**Conclusions:**

Both datasets consistently showed that Actinomyces was protective against cervical cancer (BBJ OR = 0.52; EBI OR = 0.55), while Lachnospiraceae UCG001 increased risk (BBJ OR = 2.00; EBI OR = 1.91), with no evidence of heterogeneity or pleiotropy in these robust MR analyses.

**Supplementary Information:**

The online version contains supplementary material available at 10.1007/s12672-025-03717-3.

## Introduction

Cervical cancer ranks as the fourth most prevalent cancer among women, with an estimated 569,847 new cases and 311,365 fatalities globally in 2018, constituting a substantial health burden for women worldwide [1]. In low- and middle-income countries, the incidence and mortality rates of cervical cancer are notably elevated in older women [2, 3]. The chronic infection with high-risk subtypes of human papillomavirus (HPV) invariably precipitates the development of cervical cancer [4]. Factors associated with heightened HPV susceptibility encompass early sexual initiation, engagement in sexual activity with one or multiple high-risk partners, immunosuppression, a history of sexually transmitted infections, vulvar or vaginal dysplasia, and an absence of regular screening [5–7]. Primary treatment modalities entail surgical interventions coupled with radiotherapy and chemotherapy, whereas chemotherapy drugs in combination with bevacizumab may be employed for patients with metastatic or recurrent disease, albeit limited by high costs and challenges in widespread accessibility [8–11].

Over the past decades, research has consistently demonstrated a profound connection between gut microbiota and women's health, where an imbalance in gut microbiota has been implicated in the onset of cancer [12]. Numerous clinical investigations have underscored the correlation between microbial composition disruption and various maladies affecting the female reproductive system, including infertility [13], precancerous lesions, urinary tract infections [14, 15], and even cervical [16–18], ovarian [19], and endometrial cancers [20, 21]. These associations primarily hinge on mechanisms involving the suppression of immune responses, disturbances in hormone secretion, and alterations in cell cycle regulation [22–25]. Estrogen plays a pivotal role in the gut-vaginal microbial axis, with specific bacterial populations capable of promoting extra-ovarian estrogen production.Dysregulation of estrogen levels and gut microbial status has been linked to the development of cervical cancer [16, 18, 26].

Epidemiological investigations have consistently revealed an association between non-lactobacillus-dominant vaginal microbiota and persistent high-risk HPV infection [16, 18, 26]. Conversely, a lactobacillus-dominant vaginal microbiota has been linked to the spontaneous regression of cervical intraepithelial neoplasia within approximately 12 months, while patients who did not experience regression exhibited an elevated proportion of anaerobic bacteria [17]. Notably, anaerobic microorganisms, including Atopobium vaginae, Dialister invisus, Finegold magna, Gardnerella vaginalis, Prevotella buccalis, and Prevotella timonensis, have demonstrated carcinogenic effects [27–29]. Nevertheless, it is essential to acknowledge that the outcomes of clinical observational studies are susceptible to the influence of confounding factors such as age, environmental variables, and lifestyle factors, which may render the conclusions less precise.

To investigate the potential causal influence of gut microbiota on cervical cancer risk, we performed a two-sample Mendelian randomization (MR) analysis. Instrumental variables for gut microbial composition were derived from genome-wide association study (GWAS) summary statistics available through the European Bioinformatics Institute (EBI). Genetic association data for cervical cancer were obtained from two independent sources: a European cohort (EBI GWAS catalog) and an Asian population cohort (BioBank Japan, BBJ), enabling trans-ancestry validation of findings.

## Materials and methods

### Data sources

Host genetic variants associated with gut microbial features were derived from the largest genome-wide meta-analysis to date conducted by the MiBioGen consortium (n = 9986 individuals; 8,107,040 SNPs analyzed) [30]. The cohort predominantly comprised individuals of European ancestry (78.3%). Microbial profiling was performed through 16S rRNA gene sequencing targeting hypervariable regions (V4, V3-V4, and V1-V2), with taxonomic classification achieved using direct taxonomic binning against reference databases [31].Microbiome quantitative trait locus (mbQTL) mapping identified host genetic variants significantly associated with relative abundance of bacterial taxa (false discovery rate [FDR] < 0.05) [32]. Of 211 initially characterized microbial features, 196 taxonomically defined features (excluding 15 unclassified taxa) were retained for downstream analysis. Taxonomic resolution followed standard phylogenetic hierarchy: phylum → class → order → family → genus → species, with analyses primarily conducted at the genus level (n = 131 genera with mean abundance > 1%) [30]. Genetic association statistics for cervical cancer were obtained from two independent genome-wide association studies (GWAS) in East Asian populations. The primary dataset was derived from BioBank Japan (BBJ), comprising 90,336 adult women (605 cases and 89,731 controls) of East Asian ancestry. Validation analyses incorporated summary statistics from the European Bioinformatics Institute (EBI) GWAS Catalog, which included 61,581 East Asian women (967 cases and 60,614 controls) [33].

### Instrumental variables (IVs)

Instrumental Variable Selection Criteria: The Mendelian randomization (MR) analysis employed strict criteria for instrumental variable (IV) selection to satisfy three core assumptions:Exposure Association: Genetic variants were required to exhibit genome-wide significant associations with the exposure (P < 1.0 × 10⁻⁸), ensuring sufficient predictive strength (F-statistic > 10). Confounder Independence: We eliminated SNPs showing linkage disequilibrium (LD) (r^2^ ≥ 0.001 within 10,000 kb windows) and those with minor allele frequency (MAF) < 1% to minimize population stratification bias.Exclusion Restriction: Palindromic SNPs and those in repetitive genomic regions were excluded to prevent strand orientation errors. Allelic coding consistency between exposure and outcome datasets was verified to avoid effect estimate reversal. For retained variants, we calculated F-statistics (β^2^/SE^2^) to quantify instrument strength, excluding weak IVs (F < 10) that might introduce bias. This multi-step filtering process: Incorporated LD clumping (r^2^ threshold: 0.001), Applied MAF and HWE filters, Removed structurally ambiguous SNPs, Harmonized allele orientations [34].

### Mendelian randomization analysis approach

Our primary causal inference was derived using the inverse variance weighted (IVW) method, which provides optimal statistical power when all instrumental variables satisfy Mendelian randomization assumptions [35]. This two-sample MR framework incorporated weighted median and weighted mode estimators as complementary approaches to validate IVW findings, while MR-Egger regression served to assess potential directional pleiotropy [36]. Sensitivity analyses included:Horizontal pleiotropy evaluation using MR-PRESSO (significance threshold: P < 0.05 for outlier variants) and MR-Egger intercept tests; Heterogeneity assessment via Cochran's Q statistic (IVW and MR-Egger variants); Influence analysis through leave-one-out permutation testing to identify disproportionately influential genetic variants [37]. Microbiota-CC associations demonstrating significant heterogeneity (PQ < 0.05) or pleiotropy (PMR-Egger intercept < 0.05) underwent iterative instrument refinement. Statistical power calculations were performed using mRnd (https://cnsgenomics.shinyapps.io/mRnd/) with parameters matching our study's genetic architecture.

## Results

**Fig. 1:**
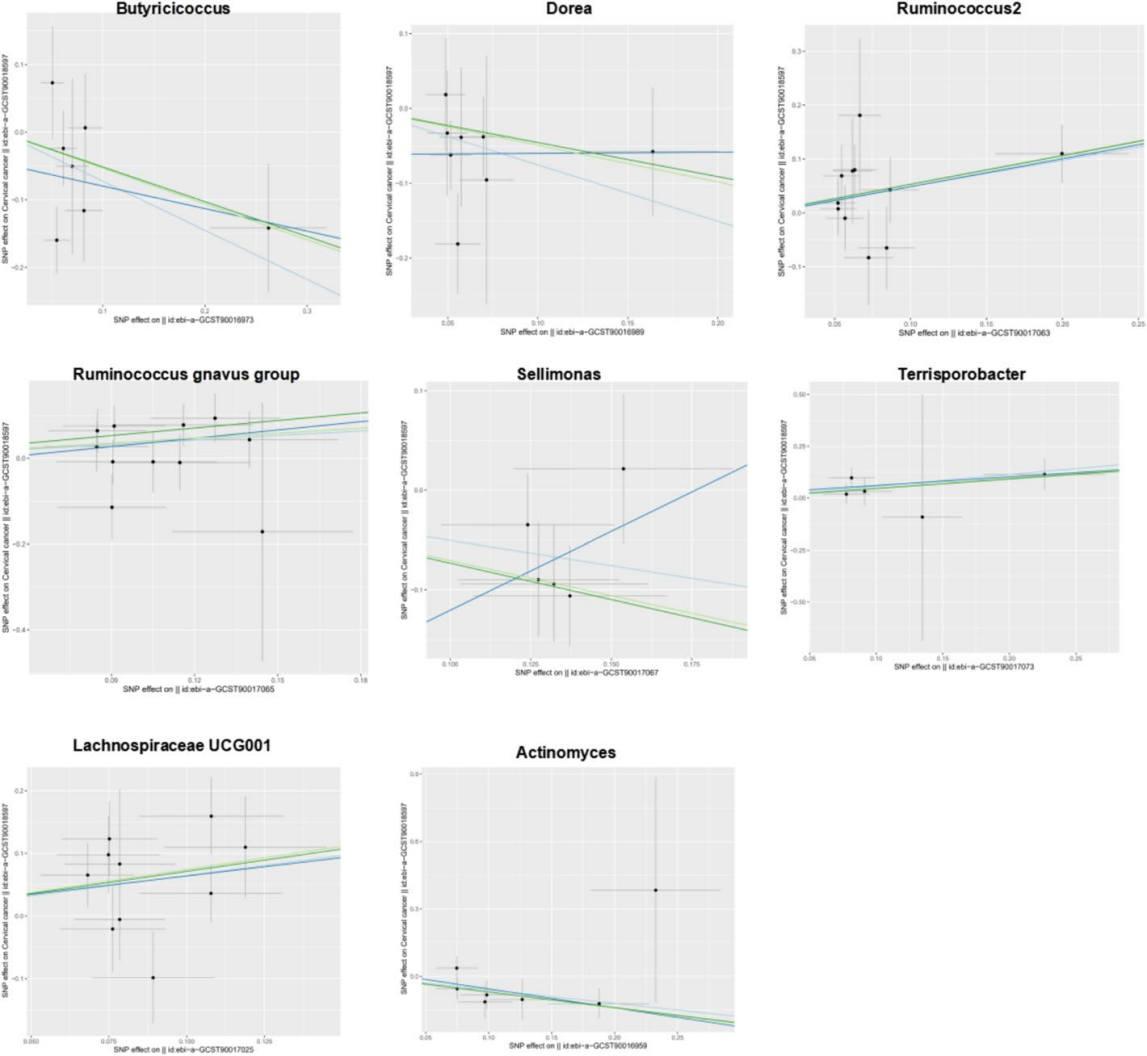
Scatter plots for the causal association between gut microbiota and CC

**Fig. 2:**
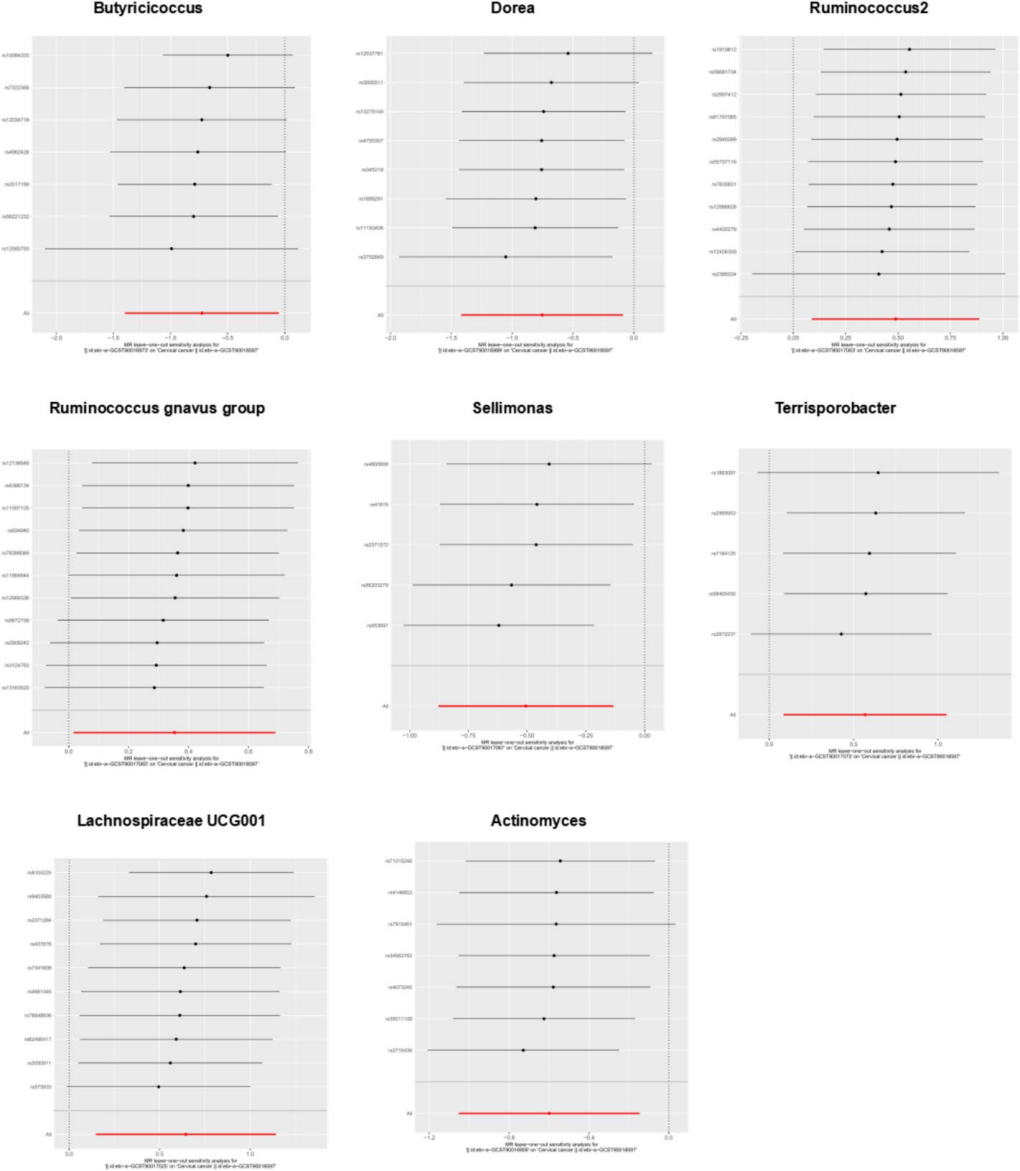
Leave-one-out plots for the causal association between gut microbiota and CC

**Fig. 3:**
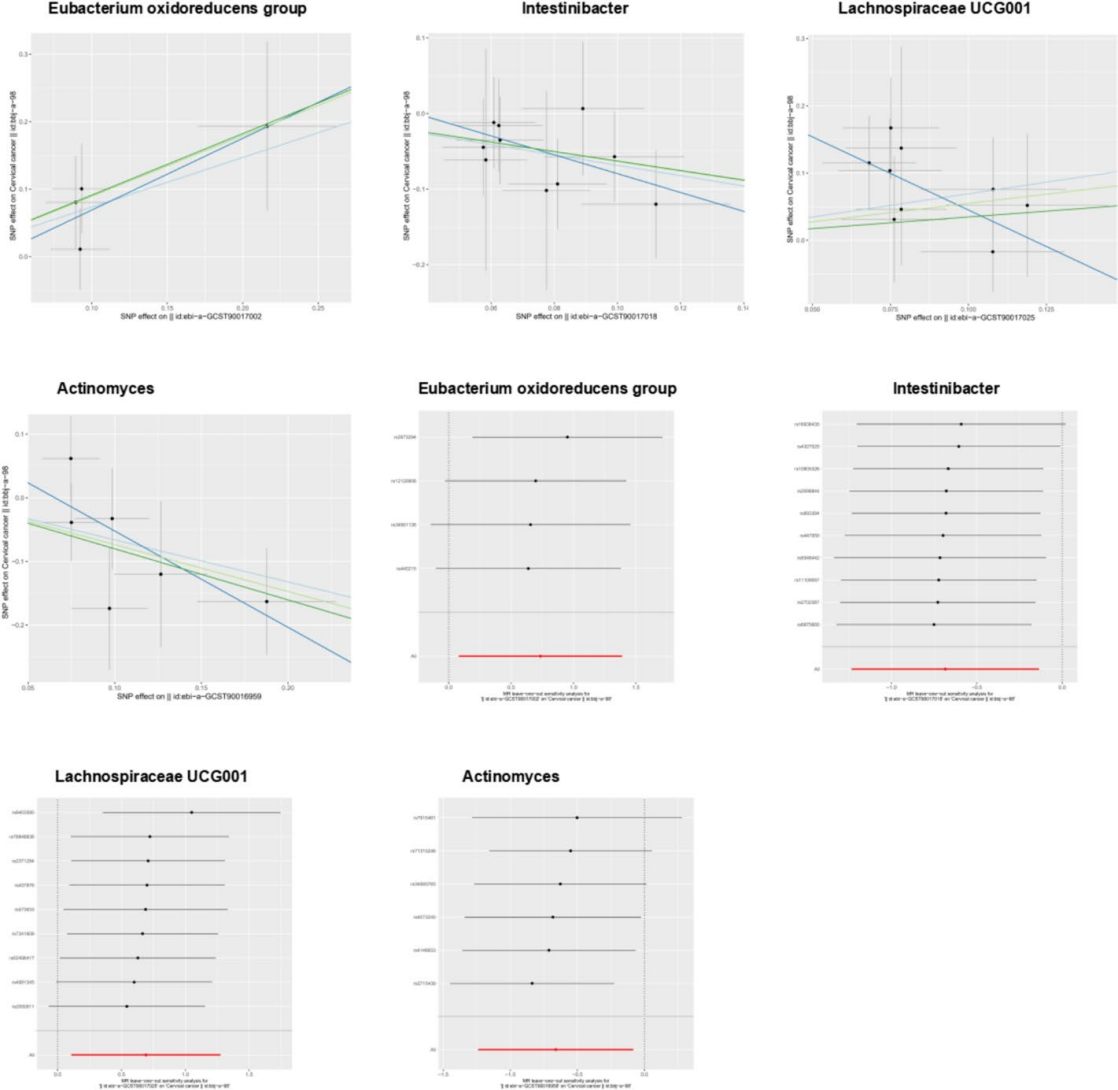
Scatter plots(Top row) and Leave-one-out plots(Bottom row) for the causal association between gut microbiota and CC

Our Mendelian randomization analysis incorporated 1609 genome-wide significant SNPs (P < 5 × 10⁻⁸) as instrumental variables across 196 bacterial genera (Supplementary Table S1), employing four complementary methods (inverse-variance weighted [IVW], MR-Egger, weighted median, and weighted mode) with BBJ cervical cancer data as the primary outcome. Initial analyses identified Intestinibacter (IVW OR = 0.50, 95% CI 0.29–0.87, P < 0.05) and Actinomyces (IVW OR = 0.52, 95% CI 1.29–0.92, P < 0.05) as showing protective effects against cervical cancer, while the Eubacterium oxidoreducens group (IVW OR = 2.08, 95% CI 1.08–4.01, P < 0.05) and Lachnospiraceae UCG001 (IVW OR = 2.00, 95% CI 1.11–3.58, P < 0.05) demonstrated risk-enhancing associations—all findings remaining significant after false discovery rate correction. These results were supported by robust instrument strength (F-statistics ranging from 85.50 to 194.91) and showed no evidence of heterogeneity (Cochran's IVW Q test P > 0.05; Supplementary Table S3) or directional pleiotropy (MR-Egger regression intercept P > 0.05; Supplementary Table S4). Validation in the EBI dataset confirmed the protective effect of Actinomyces (OR = 0.55, 95% CI 0.29–0.87, P < 0.05) and the risk-enhancing effect of Lachnospiraceae UCG001 (OR = 1.91, 95% CI 1.16–3.13, P < 0.05), while identifying additional significant taxa including Butyricicoccus (OR = 0.48, 95% CI: 0.25–0.95, P < 0.05), genus Dorea (OR = 0.47, 95% CI: 0.24–0.91, P < 0.05), Sellimonas (OR = 0.60, 95% CI 0.42-0.88, P < 0.05), Ruminococcus2 (OR = 1.63, 95% CI 1.09–1.43, P < 0.05), Ruminococcus gnavus group (OR = 1.42, 95% Cl 1.02–1.99, P < 0.05), Terrisporobacter (OR = 1.76, 95% CI 1.09–2.86, P < 0.05), and Family XIII UCG001 (OR = 2.13, 95% CI 1.23–3.70, P < 0.05). Sensitivity analyses confirmed the robustness of these findings (F-statistics > 10; Cochran's Q test P > 0.05; Supplementary Tables S6-S8), with MR-PRESSO analysis showing no evidence of horizontal pleiotropy (global test P > 0.05). The consistent results across both datasets particularly highlight the robust opposing effects of Lachnospiraceae UCG001 (promoting cervical cancer risk) and Actinomyces (exerting protective effects), with detailed instrument characteristics and complete results presented in Supplementary Tables S2-S8 and Figs. 1, 2 and 3.

## Discussion

Utilizing BBJ cervical cancer data, our analysis Discoveries bacterial taxa with potential roles in carcinogenesis. Pathogenic Bacterial Contributions: Lachnospiraceae UCG001 may exhibit dual inflammatory effects through short-chain fatty acid (SCFA) production. While physiological SCFA levels support mucosal integrity, elevated butyrate concentrations could promote localized inflammation [38, 39], potentially fostering microenvironments favoring HPV persistence and neoplastic progression [40]. Murine studies suggest certain Lachnospiraceae strains might alter Th17/Treg balance toward immune tolerance [41], which could impede viral clearance. Eubacterium oxidoreducens group metabolites, including dihydroquercetin, show dose-dependent genotoxicity in vitro [42, 43]. Its overabundance has been associated with disruption of commensal ecology, potentially displacing protective taxa like Actinomyces [44–46]. Protective Bacterial Mechanisms: Actinomyces appears to exert immunomodulatory effects by upregulating anti-inflammatory cytokines (e.g., IL-10) [47, 48], consistent with its protective role in IBD [49]. This anti-inflammatory activity may reduce HPV-related cervical damage [50]. Actinomyces-derived SCFAs could reinforce epithelial barrier function via tight junction protein induction [51, 52], potentially limiting viral integration. Competitive exclusion of pathogens like Fusobacterium further suggests its protective potential [53],though preliminary evidence indicates actinomycin metabolites might possess anti-neoplastic properties through epigenetic modulation [53].

Microbial Drivers of Cervical Carcinogenesis: Insights from EBI Cohort Analysis Using the EBI dataset, we identified microbial contributors through multi-omics integration. Ruminococcus spp. showed predominantly protective associations, though mechanisms remain incompletely characterized. Their butyrate/acetate production [54, 55] may enhance epithelial barrier function while suppressing pro-inflammatory cascades—potential mediators in HPV-related cervicitis resolution [56]. Discrepancies with cohort data could reflect strain-specific effects or uncharacterized metabolic pathways. Ruminococcus gnavus group emerged as a potential oncogenic facilitator through: Mucolytic degradation of cervical mucus [57, 58], enhancing HPV access; NF-κB activation via phenylacetic acid [59]; Immunomodulatory crosstalk enabling tumor niche formation [60, 61]; and possible molecular mimicry mechanisms [58].Terrisporobacter demonstrated genotoxic potential via: Colibactin-analog production inducing DNA breaks [62]; Acetate-driven fibroblast proliferation [63]; and bacteriocin-mediated disruption of protective Lactobacillus populations [62].

Analysis of both datasets revealed consistent patterns: Lachnospiraceae UCG001 showed significant pro-oncogenic associations, suggesting it may represent a microbial risk factor. Conversely, Actinomyces abundance correlated inversely with disease progression markers, implying protective functions. These observations suggest potential opposing roles in cervical carcinogenesis, though mechanistic understanding remains incomplete.

Our Mendelian randomization (MR) approach offers methodological strengths for establishing gut microbiota-cervical cancer causality. First, MR substantially reduces confounding biases inherent in observational studies. We derived genetic instruments from the most comprehensive gut microbiota GWAS meta-analysis available, maximizing instrument strength. Crucially, validation across homogeneous independent CC datasets (BBJ and EBI) enhanced reliability while reducing false-positive risks. The inclusion of European and Asian populations improves cross-ethnic generalizability.

Several limitations warrant careful consideration when interpreting these results. The reliance on summary-level data precluded clinical stage-specific subgroup analyses. Taxonomic resolution was constrained at the genus level, preventing species-specific causal inference. While we performed reverse MR analyses to mitigate reverse causation concerns, residual bidirectional effects cannot be entirely excluded. Although we excluded sex chromosomes and adjusted for gender, the inclusion of male participants in the microbiota GWAS may introduce unmeasured confounding. While our MR analysis provides compelling evidence for gut microbiota-CC associations, the translational implications remain fundamentally constrained by the lack of mechanistic understanding. The observed correlations—no matter how statistically robust—ultimately require validation through reductionist biological experiments before clinical application can be considered.

## Conclusions

In conclusion, this two-sample MR study found that Lachnospiraceae UCG001 and Actinomyces have causal relationships with CC that are either pathogenic or protective. Further RCT studies are needed to clarify the therapeutic or protective effects of these bacterial species on CC and their specific underlying mechanisms.While these findings offer valuable insights into microbiota-cancer associations, substantial mechanistic elucidation remains prerequisite for clinical implementation.

## Supplementary Information

Additional file 1.

## Data Availability

Gut microbiota genome-wide association study (GWAS) summary statistics were sourced from the MiBioGen consortium through the EMBL-EBI GWAS Catalog (Accessions: GCST90016908–GCST90017118; URL: https://www.ebi.ac.uk/gwas). Cervical cancer genetic associations were obtained from two independent repositories: Dataset 1 was acquired from the BioBank Japan project (BBJ; Accession: bbj-a-98; URL: https://www.bbj.ncgm.go.jp), while Dataset 2 was derived from the EMBL-EBI GWAS Catalog (Accession: ebi-a-GCST90018597; URL: https://www.ebi.ac.uk/gwas).
